# Severe combined hyperlipidaemia and retinal lipid infiltration in a patient with Type 2 diabetes mellitus

**DOI:** 10.1186/1476-511X-5-29

**Published:** 2006-12-17

**Authors:** Rachel A Davey, Niall C Tebbutt, Jenny M Favaloro, David N O'Neal, Derek Rae, Jeffrey D Zajac, James D Best

**Affiliations:** 1 Department of Medicine, University of Melbourne, Austin Health, Heidelberg, Victoria, Australia; 2 Department of Medicine, University of Melbourne, St Vincent's Hospital, Fitzroy, Victoria, Australia; 3 Department of Pathology, St Vincent's Hospital, Fitzroy, Victoria, Australia

## Abstract

Severe combined hyperlipidaemia has occasionally been associated with infiltration of tissues in addition to arteries and the skin. We report a woman with Type 2 diabetes mellitus (DM) and severe combined hyperlipidaemia who developed retinal lipid infiltration, resulting in blindness. A 61-year-old woman with a 15-year history of Type 2 DM was admitted following a two-week history of progressive visual loss. Examination identified lipid infiltration into the retina. Phenotypically she had severe combined hyperlipidaemia with elevated IDL cholesterol and a broad beta band on lipoprotein electrophoresis, raising the possibility of familial dysbetalipoproteinaemia. However, gene sequencing analysis indicated that the patient was homozygous for the E3/E3 allele of the ApoE gene with no mutations detected in either the coding region or intron-exon boundaries. Her lipid profile improved following dietary therapy and gemfibrozil treatment, but this had little effect on either her fundal appearances or her visual acuity. Type 2 DM plays a vital role both in allowing expression of severe combined hyperlipoproteinaemia, in addition to serving as a risk factor for complications such as tissue infiltration.

## Findings

Severe combined hyperlipidaemia with comparable elevation of both triglycerides and cholesterol usually has an inherited basis, such as familial dysbetalipoproteinaemia. The expression of the genetic potential for these lipid disorders is a complex process which only occurs when genetically inherited predisposing factors interact with other metabolic factors that exacerbate hyperlipidaemia. The integral genetic factor in familial dysbetalipoproteinaemia is the presence of abnormal apolipoprotein E (apoE) which binds defectively to hepatic receptors and impairs clearance of intermediate density lipoproteins (IDL) and dense very low density lipoproteins (VLDL) from the circulation [1]. Familial combined hyperlipidaemia is characterised by overproduction of apolipoprotein B (apo B) containing particles by the liver, but there is no diagnostic genetic test. Severe combined hyperlipidaemia from both these causes is associated with arterial wall lipid infiltration and atherosclerosis while familial dysbetalipoproteinaemia in particular, has been reported to cause infiltration of other tissues. This report describes a patient with Type 2 diabetes mellitus (DM) who developed a new complication of severe combined hyperlipidaemia in which lipid infiltrated into the retina, resulting in blindness.

A 61 year old woman with Type 2 DM of 15 years duration, complicated by hypertension and aortic incompetence was admitted following a 2 week history of progressive loss of vision in her left eye. The patient's Type 2 DM had been managed with diet alone, up until 6 months prior to admission when gliclazide therapy was prescribed. Home glucose monitoring revealed blood glucose levels in the range 4–9 mmol/l before meals. Other relevant past medical history included the removal of cataracts 9 months previously and a single course of laser therapy to each eye for retinopathy. Her hypertension had been well controlled by the calcium channel antagonist, felodipine. The aortic incompetence was secondary to rheumatic heart disease and culminated in a presentation with pulmonary oedema. She ultimately required an aortic valve replacement with a mechanical prosthesis 2 years before presentation. A single coronary artery graft (circumflex artery) was performed at the time, although she never suffered from any symptoms of angina. She was subsequently maintained on digoxin, frusemide and warfarin.

**Figure 1:**
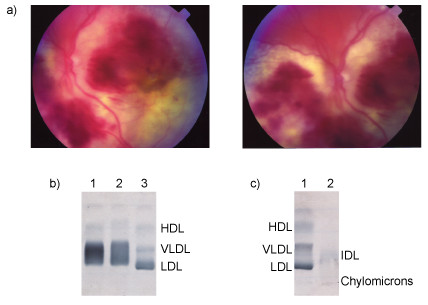
a) Photographs of the patient's eye showing gross exudative and haemorrhagic change, b) Serum lipid profile showing a broad beta band detected in the patient's serum collected on two different days; lane 1: Patient sample #1; lane 2: Patient sample #2; lane 3: control, c) Lipid profile from vitreous material obtained from patient's eye showing faint staining for Intermediate Density Lipoprotein (IDL); lane 1: control; lane 2: Patient sample.

Examination on admission revealed no evidence of any cutaneous stigmata of lipid disorders. Visual acuity was reduced to counting fingers in the left eye. Her fundal appearance in that eye was abnormal with gross exudative and haemorrhagic change (Figure 1a). Acuity in the right eye was reduced to perception of light, and fundoscopy revealed a vitreous opacity consistent with a vitreous haemorrhage.

Lipid, biochemical and haematological evaluation are outlined in Table 1. The most significant abnormalities evident were combined hyperlipidaemia and marked proteinuria (Table 1). Lipoprotein electrophoresis was performed according to the manufacturer's instructions (Beckman Coulter, CA, USA) and stained with Sudan Black 7B. A "broad beta band" was revealed, such as that found characteristically in familial dysbetalipoproteinaemia (Figure 1b). Apolipoprotein E phenotyping was determined as described previously [2]. Differentiation between the common apolipoprotein E genetic polymorphisms (apoE2, E3, E4) was performed as described by Hixson and Vernier [3]. The patient was homozygous for the E3/E3 allele of the Apo E gene. The exons and their flanking regions of the patient's ApoE gene were sequenced using Big Dye Dideoxy Terminator Chemistry (Applied Biosystems, CA, USA). All coding regions and all intron-exon splice junctions of the patient's ApoE were identical with the published sequence [4], and no ambiguities or potentially heterozygous base positions were detected. One difference however was detected in the intronic sequence of the patient compared with the published sequences (Intron A, base 69: C substituted for G) [4].

**Table 1:** Lipid, Biochemical and Haematological Evaluation.

**Lipid Profile**	**Value**	**Reference Range**
Total cholesterol	10.6 mmol/L	<5.5 mmol/l
Total triglyceride	7.5 mmol/L	<2.0 mmol/1
HDL cholesterol	0.97 mmol/L	<0.9–2.4 mmol/l
LDL cholesterol	6.6 mmol/L	<3.5 mmol/l
VLDL cholesterol	3.0 mmol/L	<0.5 mmol/l
VLDL triglyceride	5.6 mmol/L	<1.0 mmol/l
IDL cholesterol	2.9 mmol/L	
IDL triglyceride	0.8 mmol/L	
Apo A1	151 mg/dL	125–198 mg/dL
Apo B	297 mg/dL	31–151 mg/dL
ApoB/Apo A1	1.97 mg/dL	0.21–0.73 mg/dL
ApoE genotyping	E3/E3	

**Biochemical Evaluation**	**Value**	**Reference Range**

Urea	9.6 mmol/L	3.1–8.8 mmol/L
Creatinine	0.13 mmol/L	0.07–0.11 mmol/L
Albumin	31 g/L	35–50 g/L
24 hr urinary protein	3.8 g/day	0.00–0.08 g/day
Thyroid function	Normal	
Hb A1C	7.7%	<7.0%

**Haematological Evaluation**	**Value**	**Reference Range**

Haemoglobin	8.2 g/dL	11.5–16.5 g/dL
White cell count	2.4 × 10^9^/L	4.0–11.0 × 10^9^/L
Platelets	121 × 10^9^/L	150–450 × 10^9^/L
Auto antibodies	Negative	
Rheumatoid factor	Negative	
Anti nuclear factor	Negative	
Anti neutrophil cytoplasmic antibody	Negative	

The patient's proteinuria was investigated with a renal biopsy in order to exclude lipid infiltration into the kidney as this had occurred in a patient with familial dysbetalipoproteinaemia who had presented with proteinuria [5]. The biopsy showed changes consistent with diabetic nephropathy, with little evidence of foam cells to suggest a lipid infiltrative process. Haematological evaluation showed that the patient was pancytopenic (Table 1). Bone marrow aspiration and trephine biopsy revealed no evidence of marrow infiltration or aplasia, and a peripheral destructive process was thereby inferred. Since it was felt that her gliclazide might have been contributing to this process, this treatment was ceased. Subsequent blood counts showed significant resolution of the pancytopenia, with haemoglobin stabilising at 10.2 g/dL, white cell count at 3.0 × 10^9^/L and platelets at 259 × 10^9^/L, changes that coincided with improvement of her severe hyperlipidaemia.

The extent of the confluent retinal exudates and haemorrhages was felt to be significantly more severe than would be expected in typical diabetic retinopathy. A fluorescein angiogram revealed evidence of diffuse capillary leakage, but no evidence of neovascularisation. In order to investigate her retinal abnormalities further, a vitreous biopsy of the left eye was performed. This revealed a lipid infiltrate which on electrophoresis was demonstrated to be composed of IDL (Figure 1c). Since the vitreous lies anatomically adjacent to the retina, it was therefore surmised that the exudative retinal changes were secondary to an infiltration by IDL.

Further treatment included intensive dietary therapy, and subsequently gemfibrozil treatment, at a dose of 600 mg b.d. One month later, her cholesterol level was 10.9 mmol/L with triglycerides 6.6 mmol/L. Two months later she was admitted to another hospital with a separate illness. At this time, her lipid profile had improved substantially, with plasma cholesterol 5.3 mmol/L and triglycerides 2.6 mmol/L.

She had a vitrectomy performed on the right eye, but this procedure resulted in minimal improvement in her vision. Improvement in her lipid profile had little effect on either her fundal appearances or her visual acuity in the left eye.

Marked elevation of both cholesterol and triglyceride levels to above 10 mmol/L is very likely to indicate the presence of a genetic abnormality that increases hepatic production of apo B containing lipoprotein particles or decreases the metabolism of these particles, combined with a metabolic abnormality that exacerbates the inherited defect. The broad beta band on lipoprotein electrophoresis in this patient is characteristically found in familial dysbetalipoproteinaemia, but may also be present in other subjects with severe combined hyperlipidaemia. Prior to the availability of apo E phenotyping and genotyping, criteria of VLDL-cholesterol/serum triglyceride ratio >0.69 and VLDL-cholesterol/VLDL-triglyceride ratio >0.92 were used to define the presence of VLDL remnants and familial dysbetalipoproteinaemia [6,7].

In this patient the ratios did not meet these criteria and phenotyping, genotyping and gene sequencing did not show any abnormality of apo E. It is therefore not possible to sustain the diagnosis of familial dysbetalipoproteinaemia, despite the presence of a broad beta band. The findings could be consistent with severe familial combined hyperlipidaemia, which is characterised by hepatic overproduction of apo B containing particles. However, there is no specific diagnostic test for this condition.

Type 2 DM is commonly associated with dyslipidaemia, particularly elevation of VLDL triglyceride and reduction of HDL cholesterol. Overproduction of VLDL particles has been well documented and this metabolic abnormality is likely to exacerbate the combined hyperlipidaemia associated with the inherited disorders of familial dysbetalipoproteinaemia and familial combined hyperlipidaemia. Diabetes has been suggested as a prominent cofactor for the development of familial dysbetalipoproteinaemia for a number of years. Studies such as those by Eto et al [8] are in accordance with the proposal that Type 2 DM is a prominent risk factor for the development of elevated lipid levels in patients with apoE2/E2. Furthermore, in the present case, the heavy proteinuria which occurred as a result of diabetic nephropathy is also likely to have played a significant role as a cofactor for the expression of severe combined hyperlipidaemia. Proteinuria is associated with hyperlipidaemia when it reaches nephrotic range and in this case may be a consequence of excess hepatic VLDL secretion. Therefore, the Type 2 DM is likely to have had a dual role in the expression of the combined hyperlipidaemia.

Infiltration of arterial wall tissue by lipoproteins, causing atherosclerosis and vessel narrowing, is a common complication of severe combined hyperlipidaemia. Infiltration of dermal tissue by lipids causes xanthomata and is well described as occurring in the combined hyperlipidaemia associated with familial dysbetalipoproteinaemia. There have also been reports of lipid deposition in kidney, splenic and bone marrow tissue in this condition [5,9]. Deposition of lipoproteins in retinal tissue is very unusual and in this patient occurred in the setting of marked elevation of cholesterol and triglyceride, carried in relatively dense VLDL particles and IDL particles, but not associated with abnormal apo E.

Type 2 DM may have also played a role in the retinal infiltration of lipids seen in this patient. Lipid accumulation in renal glomeruli has been previously described in patients with familial dysbetalipoproteinaemia who were known to have Type 2 DM or who were diagnosed with Type 2 DM within six months and presented with proteinuria [5,9]. Furthermore, reduction of serum lipids with lipid lowering drugs in patients with Type 2 DM, diabetic maculopathy and hyperlipidaemia has been shown to result in a dramatic regression of retinal hard exudates [10]. In contrast, improvement in the current patient's lipid profile had little effect on the fundal appearance or visual acuity of the eye. Lipid infiltration into the retina associated with severe combined hyperlipidaemia however, is extremely unusual. It seems highly likely that one of the principal causes of lipid accumulation in the retina is the increased endothelial permeability that is associated with microvascular disease in diabetic patients. The haemorrhagic changes in the retina that were observed in this case are likely to have arisen from the combination of increased endothelial permeability and anticoagulation with warfarin.

An additional contributing factor to the pathogenesis in this case could also be abnormal lipid metabolism, thereby increasing the patient's susceptibility to forming lipid deposits within the eye. It has recently been demonstrated that lipoproteins isolated from the Bruch's membrane differ significantly from plasma lipoproteins and that the genes for apoB and apoA-I are expressed within the eye [11]. These data suggest that the retinal pigment epithelium assembles and secretes large alipoprotein particles. Furthermore, studies in mice over-expressing ApoB suggest that hyperlipidemia predisposes mice to the formation of basal laminar deposits by altering hepatic and/or retinal pigment epithelium lipid metabolism [12].

This case is unusual in a number of respects. Firstly, it represents an uncommon complication of any lipid disorder, with tissue infiltration by lipid. Secondly, despite a phenotype that resembled familial dysbetalipoproteinaemia, the ApoE genotype of this patient was E3/E3, and not the E2/E2 genotype associated with this disease. Furthermore, this case emphasises how Type 2 DM and associated proteinuria can act as prominent metabolic cofactors to exacerbate combined hyperlipidaemia and precipitate tissue infiltration.

## Competing interests

The author(s) declare that they have no competing interests.

## Authors' contributions

RAD, NT, JDZ and JDB have been involved in drafting the manuscript and revising it critically for important intellectual content, NT and JDB were responsible for assessing the patient, JMF was responsible for the genotyping analyses, DON and DR were responsible for the biochemical analyses.
